# Contextualising Safety in Numbers: a longitudinal investigation into change in cycling safety in Britain, 1991–2001 and 2001–2011

**DOI:** 10.1136/injuryprev-2017-042498

**Published:** 2017-11-30

**Authors:** Rachel Aldred, Rahul Goel, James Woodcock, Anna Goodman

**Affiliations:** 1 Department of Planning and Transport, faculty of Architecture and the Built Environment, Westminster University, London, UK; 2 UKCRC Centre for Diet and Activity Research (CEDAR), MRC Epidemiology Unit, University of Cambridge School of Clinical Medicine, Cambridge, UK; 3 Department of Population Health, London School of Hygiene & Tropical Medicine, London, UK

**Keywords:** bicycle, cross sectional study, time series

## Abstract

**Introduction:**

The ’Safety in Numbers’ (SiN) phenomenon refers to a decline of injury risk per time or distance exposed as use of a mode increases. It has been demonstrated for cycling using cross-sectional data, but little evidence exists as to whether the effect applies longitudinally —that is, whether changes in cycling levels correlate with changes in per-cyclist injury risks.

**Methods:**

This paper examines cross-sectional and longitudinal SiN effects in 202 local authorities in Britain, using commuting data from 1991, 2001 and 2011 censuses plus police -recorded data on ’killed and seriously injured’ (KSI) road traffic injuries. We modelled a log-linear relationship between number of injuries and number of cycle commuters. Second, we conducted longitudinal analysis to examine whether local authorities where commuter cycling increased became safer (and vice versa).

**Results:**

The paper finds a cross-sectional SiN effect exists in the 1991, 2001 and 2011 censuses. The longitudinal analysis also found a SiN effect, that is, places where cycling increased were more likely to become safer than places where it had declined. Finally, these longitudinal results are placed in the context of changes in pedestrian, cyclist and motorist safety. While between 1991 and 2001 all modes saw declines in KSI risk (37% for pedestrians, 36% for cyclists and 27% for motor vehicle users), between 2001 and 2011 pedestrians and motorists saw even more substantial declines (41% and 49%), while risk for cyclists increased by 4%.

**Conclusion:**

The SiN mechanism does seem to operate longitudinally as well as cross-sectionally. However, at a national level between 2001–11 it co-existed with an increase in cyclist injury risk both in absolute terms and in relation to other modes.

## Introduction

The ‘Safety in Numbers’ (SiN) phenomenon refers to a decline in injury risk as use of a mode increases. This type of effect was identified by Smeed^1^ in relation to motor vehicles, that is, an inverse relationship between a country’s level of motorisation and road deaths per motor vehicle. Recent work has focused on walking and cycling, often prompted by a desire to increase active travel, which can substantially benefit population health.^2^ However, cyclists and pedestrians are particularly vulnerable to serious injury arising from collision with motorised vehicles, and fear of such injury remains a key barrier to uptake, particularly for cycling.

Work on walking and cycling has shown a SiN effect in a range of contexts and at different scales. Cross-national comparisons (eg, refs 3 and 4) highlight the contrast between countries with high cycling levels and lower injury rates per cyclist (eg, the Netherlands, Denmark), and those with low cycling levels and higher injury rates per cyclist (eg, UK, USA). Such gaps would appear even larger stratified by age, because high-cycling countries tend to have a high proportion of older cyclists with increased vulnerability to injury.^5^

SiN-type differences are likewise found in within-country comparisons. In New Zealand, Tin *et al* described^6^ ‘risk in scarcity’, with lower levels of cycling associated with cycling being riskier. A recent systematic review and meta-analysis^7^ found a surprisingly consistent SiN effect across studies. However, the authors were only able to include in their meta-analysis one area-level study (as opposed to junction or road link-level analysis)that both used robust statistical methods and controlled for motor traffic volumes.^8^

Specific mechanisms for the observed SiN effects remain under discussion.^9^ Many authors referenced above suggest driver behaviour is better where there are more cyclists. Drivers may be more likely to cycle, to know people who cycle or be more used to seeing cyclists while driving, so they are more attuned to looking for them.^10^ While plausible, these are not the only potential explanations. Higher cycling cities and countries may have better cycling infrastructure and other policies, which may keep cyclists safer than places with less cycling (where there is less pressure to improve infrastructure). Alternatively, high ‘bicycle density’ on specific roads might mean each individual cyclist is less exposed to each motor vehicle.^9^

Whatever the precise mechanism, policymakers hope that if contexts with low cycling and relatively high risks (such as Britain) can increase cycling levels—for example, through improved infrastructure—this will be accompanied by a decrease in risk and hence a less than proportional increase in injuries. Most previous studies are cross-sectional, however, and one cannot assume that the effects found cross-sectionally will hold true longitudinally.^11^ In other words, even if we know that places with more cycling are also safer, do places where cycling increases become safer (or, given the potential for reverse causation, do places where cycling is becoming safer see more cycling)?

This paper examines SiN in Britain using longitudinal data, specifically commuting data from 1991, 2001 and 2011 censuses combined with police-recorded road traffic injury data. While overall cycling rates changed little during this period, there was substantial variation, with some authorities seeing growth and others decline. To our knowledge, this is the first time that a SiN analysis has simultaneously presented cross-sectional and longitudinal results. This also allows a comparison between the operation of SiN and overall changes in cycling risk. Finally, we contextualise our analysis by comparing total changes in cycling risk over time with changes in risks experienced by motorists and pedestrians.

## Methods

### Census data on main commute mode

The UK Census happens every 10 years and is compulsory for all residents (data available from https://www.nomisweb.co.uk/). In Britain (England, Scotland and Wales, the three UK countries for which suitable data are available), the estimated proportion of people covered was 96% in 1991, 94% in 2001 and 94% in 2011.^12 13^ In the 2001 and 2011 censuses, all respondents aged 16–74 with a current job were asked ‘How do you usually travel to work? (Tick one box only, tick the box for the longest part, by distance, of your usual journey to work)’. The same question was asked in 1991 of random 10% sample of respondents. One of the response options was ‘bicycle’. People working from home were not counted as commuters.

With its exceptional population coverage (completing the survey being a legal requirement), the Census is particularly suitable for exploring participation in minority modes at the local level.^i^ One disadvantage is that it does not capture occasional commuter cycling, multimodal commuter cycling or cycling for non-commuting purposes. Yet, although this focus on ‘usual main mode’ means the Census directly measures only a third of adult cycling, at a population level this provides a good proxy for total levels of cycling among residents of an area.^14^ This population-level correlation is also high in relation to public transport and motor vehicle use.

### Department for Transport estimates of motor vehicle kilometres

To estimate the volume of motor vehicle traffic, we used estimates of motor vehicle kilometres per year per local authority, produced by the UK Department for Transport as part of the road traffic series (data available from https://www.gov.uk/government/statistical-data-sets/tra89-traffic-by-local-authority, table TRA8904). These estimates are generated using data from around 8000 roadside 12-hour manual counts and continuous data from around 300 automatic traffic counters, alongside data on road lengths.^15^ We used these estimates from the years 1993, 2001 and 2011: we used 1993 instead of 1991 as this was the first year for which this data series is available.

### Police data on road traffic fatalities and serious injuries

The Stats19 data set records routinely collected police data on road traffic fatalities and injuries in Britain (data available from https://data.gov.uk/dataset/road-accidents-safety-data). This data set covers RTCs involving at least one vehicle (a bicycle counts as a vehicle) occurring on the public highway. Thus, casualties recorded in Stats19 include both drivers and passengers of vehicles, plus any pedestrians hit. Casualties include incidents involving just a single vehicle, for example a driver losing control of their car or a cyclist falling off their bicycle, without any other vehicle involved.

In this paper, we focused on fatalities (defined in Stats19 as dying within 30 days) and serious injuries (defined in Stats19 as admitted to hospital as an inpatient, and/or a list of other injuries such as fractures). This is typical in road injury research, with this category referred to as ‘KSI’ for ‘Killed or Seriously Injured’. Using KSIs rather than only fatalities provides much greater statistical power and helps reduce the impact on the results of improved trauma care over time, which all else being equal would mean some former deaths would become serious injuries. To further achieve stable KSI counts, we used Stats19 data from the 3-year periods 1990–1992 for comparison with the 1991 Census, 2000–2002 for comparison with the 2001 Census, and 2010–2012 for comparison with the 2011 Census.

Stats19 data do not include injuries away from the highway network, but do include injuries on footways or on cycle tracks adjacent to the carriageway. This may particularly under-represent injuries to cyclists, although this is less important for transport cycling, as injury risks on off-road infrastructure are substantially lower than on-road risks.^16^ Entirely off-highway infrastructure is also likely to be disproportionately used for leisure riding, another reason why Stats19 is a reasonable source for analysis comparing KSI numbers with cycle commuting numbers. In Stats19, deaths are well reported, serious injuries somewhat less so, and slight injuries much less so.^17^

### Statistical analyses

Our units of analyses were 202 areas in Britain. These were created from 206 local authorities (county-unitary level) after following the common practice of merging the two very small local authorities of the City of London (a local authority within Greater London) and Isles of Scilly with Westminster and Cornwall, respectively. We additionally merged Merthyr Tydfil with neighbouring Caerphilly, and Orkney with nearby Shetland, due to zero cycling KSI counts (which could not be entered into our longitudinal models) in some years in these two very small local authorities. The mean population for the 202 areas in 2011 was 303 818 (SD=248 682) and the median was 234 172. The smallest population was 27 684 and the largest 1 463 740.

Cross-sectional injury prediction models typically^7^ have a log-linear form and in absolute terms are expressed as the following:

$$CycleKSI\;=\alpha \times \;{\left(Cycle\;volume\right)}^{\beta 1}\;\times \;\;{\left(MV\;volume\right)}^{\beta 2}$$

We implemented this equation in two ways: first as shown in equation 1a, and second after additionally including a term for population in the model. The advantage of the first approach is that it is more directly comparable with most published studies. The advantage of the second approach is that it adjusts for the considerable differences in size between local authorities in Great Britain. This adjustment makes it possible to distinguish between local authorities with a comparatively large number of cyclists because cycling is common in that local authority (which might be expected to confer a SiN effect) versus local authorities with a comparatively large number of cyclists because the local authority was considerably larger than any of its neighbours (ie, an issue of administrative boundaries, which would not in itself be expected to confer any SiN effect).

Thus the following were the equations we implemented for 2011:

(1a) $$\begin{array}{ll} CycleKSI\;(2010-12)= & \alpha \times (CycleCommuters\_2011{)}^{\beta 1}\\ & \times (MVVolume\_2011{)}^{\beta 2} \end{array}$$

(1b) $$\begin{array}{ll} CycleKSI(2010-12)= & \alpha \times (CycleCommuters\_2011{)}^{\beta 1}\\ & \times (MVVolume\_2011{)}^{\beta 2}\times (Population\_2011{)}^{\beta 3} \end{array}$$

where ‘α’ is a constant, ‘$CycleKSI_{2010-12}$’ is the expected number of cycling KSI 2010–2012, ‘$CycleCommuters_{2011}$ is the number of commuters cycling in 2011, ‘$MVVolume_{2011}$’ is the estimated annual motor vehicle kilometres in 2011, and ‘$Population_{2011}$’ is the total population in 2011. The SiN hypothesis is that the coefficient ‘β1’ is less than 1, that is, the number of KSI increases more slowly than the number of cycle commuters. To test this, we modelled equation 1a and b in the log-linear form using generalised linear modelling (GLM) with negative binomial distribution as presented in equation 2a and b. Use of negative binomial models is typical in injury counts model to account for overdispersion.^18^

(2a) $$\begin{array}{ll} In(CycleKSI(2010-12))= & \alpha +\beta 1\times In(CycleCommuters\_2011)+\beta 2\\ & \times In(MVVolume\_2011) \end{array}$$

(2b) $$\begin{array}{ll} In(CycleKSI(2010-12))= & \alpha +\beta 1\times In(Cycle\times Commuters\_2011)\\ & +\beta 2\times In(MVVolume\_2011)\\ & +\beta 3\times In(Population\_2011) \end{array}$$

We fit equivalent models for the cross-sectional analyses of 1991 and 2001. We also fit multilevel repeated measures negative binomial models, which included data from all three time points, in a hierarchical structure of years nested within local authorities. These models were fitted as mixed effects using fixed effects for the three covariates and a random intercept term for the local authority.

For longitudinal analyses, we examined the association between the relative change in the absolute numbers of cycle commuters and cycle KSIs—that is, to answer the question ‘If the number of cycle commuters doubled, did the number of cycle KSIs double too?’ If it less-than-doubled, we took this as being compatible with a SiN effect. The injury prediction models we sought to fit were adapted versions of equation 2a and b:

$$\Delta CycleKSI=\alpha \times (\Delta CycleCommuters{)}^{\beta 1}\times (\Delta MVVolume{)}^{\beta 2}$$

(3a) $$\begin{array}{ll} In(\Delta CycleKSI)= & \alpha \times \beta 1\times In(\Delta CycleCommuters)\\ & +\beta 2\times In(\Delta MVVolume) \end{array}$$

$$\begin{array}{ll} \Delta CycleKSI= & \alpha \times (\Delta CycleCommuters{)}^{\beta 1}\\ & \times (\Delta MVVolume{)}^{\beta 2}\times (\Delta Population{)}^{\beta 3} \end{array}$$

(3b) $$\begin{array}{ll} In(CycleKSI)= & \alpha \times \beta 1\times In(\Delta CycleCommuters)\\ & +\beta 2\times In(\Delta MVVolume)\\ & +\beta 3\times In(\Delta Population) \end{array}$$

In equation 3a and b ‘α’ is again a constant, and ‘ΔCycleKSI’ is the relative change across the study period in the number of cycle KSIs, calculated as the ratio ‘CycleKSI2010-2012/CycleKSI2000-2002’. Similarly, ΔCycleCommuters is the relative change across the study period in the number of commuters cycling (calculated as ‘CycleCommuters_2011_/CycleCommuters_2001_’); ΔMVVolume is the relative change in the number of motor vehicle kilometres. Thus, while equation 2a and b relate the number of injuries with the number of cyclists, equation 3a and b relate the relative change in the number of injuries with the relative change in the number of commuter cyclists. Unlike equation 2a and b with counts of injuries, the dependent variable in equation 3a and b is a continuous variable. Therefore, we fit the model presented in equation 3a and b using GLM with Gaussian distribution, and a log link. All analyses were conducted using Stata V.14.1.

The Stata code and data tables are included in the online supplementary appendices.

### Sensitivity analyses

In sensitivity analyses, we excluded the 32 local authorities in London, because commuting between the 33 local authorities is spatially unbalanced. Central boroughs such as City of London and Westminster have low (for Inner London) levels of cycle commuting among residents, yet high cycling flows, because people living further out cycle into those central boroughs to access jobs and services. We also conducted sensitivity testing excluding ‘no other vehicle’ cycle collisions. Both sets of sensitivity analyses produced similar results to the main models described below.

## Results

### National trends in commuting and KSI trends between 1991, 2001 and 2011

As shown in table 1, nationally the number of commuters went up by 3% between 1991 and 2001, and increased by a further 10% between 2001 and 2011. Over the same period, the number of cycle commuters declined by 4% between 1991 and 2001, but then increased by 14% between 2001 and 2011. The result was that the proportion of commuter cycling fell from 3.1% in 1991 to 2.9% in 2001, and then rose to 3.0% in 2011. Over the same period, there were modest declines in the proportion of commuters walking and using motor vehicles.

**Table 1:** Descriptive statistics for Britain

		1991	2001	2011	Relative change (%) 1991–2001	Relative change (%) 2001–2011
Travel	Commuters (n)	22 860 406	23 496 787	25 943 690	+2.8	+10.4
Behaviour	Commuters cycling (n)	709 484	681 831	775 204	−3.9	+13.7
	% Commuters cycling	3.1	2.9	3.0	−6.5	+3.0
	Commuters walking (n)	2 836 709	2 618 039	2 835 170	−7.7	+8.3
	% Commuters walking	12.4	11.1	10.9	−10.5	−1.9
	Commuters using motor vehicles* (n)	17 861 048	18 313 956	19 759 587	+2.5	+7.9
	% Commuters using motor vehicles	78.1	77.9	76.2	−0.3	−2.3
Injuries	KSI (n)	175 329	121 531	74 326	−30.7	−38.8
	KSIs among cyclists (n)	12 781	7898	9303	−38.2	+17.8
	% KSIs among cyclists	7.3	6.5	12.5	−10.9	+92.6
	KSIs among pedestrians (n)	46 579	27 193	17 491	−41.6	−35.7
	% KSIs among pedestrians	26.6	22.4	23.5	−15.8	+5.2
	KSIs among motor vehicle users (n)	112 318	83 721	46 108	−25.5	−44.9
	% KSIs among motor vehicle users	64.1	68.9	62.0	+7.5	−10.0
Injuries per commuter	KSIs per 1000 commuters, cyclists	18.0	11.6	12.0	−35.7	+3.6
	KSIs per 1000 commuters, pedestrians	16.4	10.4	6.2	−36.7	−40.6
	KSIs per 1000 commuters, motor vehicle users	6.3	4.6	2.3	−27.3	−49.0

*Motor vehicle use includes travelling by car, van, taxi or motorcycle, as a driver or passenger, or travelling by bus.

KSI, killed or seriously injured.

With regard to KSI injuries, table 1 indicates that the number of KSI among cyclists decreased by 38% between 1991 and 2001, but then increased by 18% between 2001 and 2011. By contrast, there was a continued trend between 1991–2001 and 2001–2011 for the number of KSIs to decrease substantially for pedestrians and motor vehicle users. The result was that, expressing these injuries in terms of number of KSIs per thousand commuters, declines in risk of a similar scale were observed between 1991 and 2001 for cyclists, pedestrians and motor vehicle users (range 27%–37% decrease). By contrast, between 2001 and 2011 risk per commuter increased by 4% for cyclists, whereas it continued to decrease by 41%–49% for pedestrians and motor vehicle users.

### SiN at the local authority level: changes in absolute numbers of cycle commuters and cycling KSI

#### Descriptive statistics

Table 2 and table 3 compare changes in KSI risk per commuter between 1991 and 2001, and 2001 and 2011, in relation to authorities where the percentage of cycle commuters both grew and fell. In both years, those authorities where the percentage of cycle commuters grew were more likely to see a decline in cyclist risk. Apart from this, however, the pictures for 1991–2001 and 2001–2011 are very different. In 1991–2001 cyclists in the large majority (93%) of authorities enjoyed a reduction in risk, but in 2001–2011 this was true of only 50% of local authorities, with the other 50% showing an increase in risk per commuter. By contrast, reductions in risk per commuter were seen in the large majority of all local authorities in both time periods (97% for pedestrians in 1991–2001, and 95% for pedestrians in 2001–2011; 86% for motor vehicle users in 1991–2001, and 99% for motor vehicle users in 2001–2011).

**Table 2:** Changes in commuting and killed or seriously injured (KSI) by authority, 1991–2001

	Authorities where cycle KSIs per commuter fell, 1991–2001	Authorities where cycle KSIs per commuter grew, 1991–2001	All
Authorities where cycle commuting (as %) declined, 1991–2001	95 (90%)	11 (10%)	106
Authorities where cycle commuting (as %) increased, 1991–2001	93 (97%)	3 (3%)	96
All	188 (93%)	14 (7%)	202

**Table 3:** Changes in killed or seriously injured (KSI) and commuting by authority, 2001–2011

	Authorities where cycle KSIs per commuter fell, 2001–2011	Authorities where cycle KSIs per commuter grew, 2001–2011	All
Authorities where cycle commuting (as %) declined, 2001–2011	54 (44%)	69 (56%)	123
Authorities where cycle commuting (as %) increased, 2001–2011	48 (61%)	31 (39%)	79
All	102 (50%)	100 (50%)	202

Therefore, despite the SiN effect, KSI risk per cyclist grew overall between 2001 and 2011, while falling for other modes. One reason is that cycle commuting tended to grow between 2001 and 2011 in places that were riskier in 2001, and SiN did not negate this. Places where cycle commuting grew between 2001 and 2011 had 31% more KSIs per commuter in 2001 than those where it did not. This trend was even stronger between 1991 and 2001 (places where cycle commuting grew between 1991 and 2001 had double the KSIs per commuter in 1991 than those where it did not); however, the overall decline in cycling risk made up for the shift of cycling into riskier places.

#### Cross-sectional models

Table 4 presents the results of fitting regression equations 2a and 2b (cross-sectional analyses, with and without adjustment for population size). As it shows, before adjusting for population size (model A), the cross-sectionalβ coefficient for the association between the number of cycling commuters and the number of cycling KSI was 0.44 (95% CI 0.37 to 0.51) in 1991, 0.53 (0.42 to 0.57) in 2001 and 0.60 (0.52 to 0.68) in 2011. Hence at all three time points, a greater number of cycle commuters was associated with a greater total number of cycling KSI (because the coefficient is greater than 0), but the number of KSI increased more slowly than the total number of cyclists (because the coefficient is less than 1). After adjusting for population size (model B), the β coefficients became somewhat smaller, that is, corresponding to a larger SiN effect.

**Table 4:** Cross-sectional models, with and without adjustment for population (n=202)

	Cross-sectional 1991		Cross-sectional 2001		Cross-sectional 2011		Repeated measures cross-sectional (1991, 2001, 2011)	
	Model A	Model B	Model A	Model B	Model A	Model B	Model A	Model B
Cycle commuters	0.48 (0.41 to 0.55)	0.42 (0.35 to 0.48)	0.68 (0.60 to 0.76)	0.52 (0.45 to 0.60)	0.75 (0.69 to 0.82)	0.62 (0.54 to 0.70)	0.61 (0.55 to 0.67)	0.47 (0.42 to 0.53)
Motor vehicle kilometres	0.30 (0.20 to 0.40)	−0.13 (−0.27 to 0.01)	0.14 (0.05 to 0.23)	−0.32 (−0.46 to –0.19)	0.15 (0.07 to 0.22)	−0.10 (−0.22 to 0.02)	0.16 (0.08 to 0.24)	−0.24 (−0.34 to –0.14)
Population		0.69 (0.51 to 0.86)		0.86 (0.67 to 1.05)		0.50 (0.29 to 0.71)		0.76 (0.62 to 0.91)

Model A implements equation 2a, model B implements equation 2b: the difference between the two is that model B additionally adjusts for the local authority population size.

These results are therefore compatible with a cross-sectional ‘Safety in Numbers’ effect at all time points, with the effect being particularly marked after the confounding effect of population size was taken into account. There was, however, evidence that the magnitude of the SiN effect was weakening over time (P<0.001 for trend towards a larger coefficient over time in both model A and model B, as judged by fitting a linear interaction term between year and the cycle commuter term in the model).

Without adjustment for population size (model A), we found evidence that a higher volume of motor vehicle kilometres was associated with a higher number of cycling KSI. After adjusting for population size, however, the direction of this effect reversed to be a negative trend in all 3 years (although it only reached statistical significance in 2001 and in the combined repeated-measures model). This suggests that the positive effects observed in model A were due to confounding by population size (ie, on average a large local authority will have a higher total motor vehicle volume and a larger number of cycling KSI).

#### Longitudinal models

Table 5 shows that in longitudinal analysis not adjusting for population size (model A), we found a β coefficient for cycling of 0.33 (0.10 to 0.56) for the association between the relative change (ratio) in the number of commuter cyclists and the relative change in the number of cycling KSI in 1991–2001. The results after adjusting for relative change in population were very similar. These regression coefficients were greater than 0 but less than 1, compatible with a longitudinal ‘Safety in Numbers’ effect.

**Table 5:** Longitudinal models, with and without adjustment for population (n=202)

	Relative change in cycle KSI, 1991–2001		Relative change in cycle KSI, 2001–2011	
	Model A	Model B	Model A	Model B
Relative change in cycle commuters	0.33 (0.10 to 0.56)	0.34 (0.11 to 0.57)	0.79 (0.50 to 1.08)	0.75 (0.42 to 1.08)
Relative change in motor vehicle kilometres	−1.14 (−2.35 to 0.08)	−1.16 (−2.39 to 0.07)	2.15 (0.87 to 3.43)	2.19 (0.89 to 3.49)
Relative change in population		−0.13 (−0.75 to 0.50)		0.45 (−1.19 to 2.08)

Model A implements equation 3a, model B implements equation 3b: the difference between the two is that model B additionally adjusts for the local authority population size.

KSI, killed or seriously injured.

Between 2001 and 2011, the β coefficient for cycling was 0.79 (0.50 to 1.08) in model A and 0.75 (0.42 to 1.08) in model B. The 95% CI therefore included 1 in this time period, providing less clear evidence that any longitudinal safety numbers effect was operating.

In both model A and model B, the coefficient for change in motor vehicle kilometres showed a non-significant negative trend between 1991 and 2001 (0.06≥P≥0.07). By contrast between 2001 and 2011, there was strong evidence of the larger relative increase in motor vehicle kilometres being associated with the relative increase in the number of cycle KSI (both P<0.001).

## Discussion

Considering absolute cycling safety at the local level, our results support the existence of the SiN effect both cross-sectionally (relating the absolute number of cyclists to the number of cycling KSI) and are consistent with a SiN effect longitudinally (relating the relative change in the number of cyclists to the relative change in the number of cycling KSI). Both cross-sectionally and longitudinally, evidence for SiN is weaker in more recent time periods.

A less optimistic picture emerges considering trends in cycling safety nationally, and relative to other modes, in the second decade covered. It is concerning that the absolute numbers of KSIs per commuter cyclist increased in 50% of all local authorities between 2001 and 2011, whereas a comparable increase was observed for only 5% of British local authorities in respect to walking and 1% in respect to motor vehicle use. Even for those local authorities where cycling increased, two in five (39%) saw cycling becomes riskier between 2001 and 2011. This was marked a change from 1991 to 2001, where 93% of authorities saw a decline in cyclist KSI risk per commuter.

Therefore, despite the SiN effect, KSI risk per cyclist grew overall between 2001 and 2011, while falling for other modes. As a result, across the full time period 1991 and 2011, cycling became relatively riskier compared both with motor vehicle use and walking. This demonstrates that at a national level, SiN can coexist with a decline in cycle safety even alongside a small rise in cycling levels, as in 2001–2011.

We found counterintuitive results for motor vehicle use cross-sectionally (but not longitudinally) when adjusting for population: higher levels of motor vehicle use being associated with lower levels of cycle KSIs. One possible explanation might be that the apparent negative effect of motor vehicle volume could be an indicator of higher congestion levels and, therefore, higher safety of cyclists. In any case we would recommend more research attempting to separate out the impact of population from cycle and motor vehicle use, as previous studies may have been confounded by population. This could involve using smaller geographies and including a measure of congestion/motor vehicle speeds.

### Strengths and limitations

This paper relies on secondary analysis of Census, Department for Transport and Stats19 data. We believe all three data sets are adequate for our purposes. One advantage of these data sets is their total population coverage and our consequent ability to increase study power by examining SiN across many relatively small areas. The data are available across many years, allowing us to include a longitudinal aspect typically missing from SiN analysis. Nevertheless, as outlined in the methods section, there are limitations. The Census only measures changes in commuter cycling and Stats19 substantially underestimates absolute numbers of road traffic injuries. If 2001–2011 coverage of cyclist KSIs in Stats19 improved relative to other modes, this might bias our findings. However, the Department for Transport^19^ suggests this is not the case based on comparisons for Hospital Episode Statistics data, which show related trends to those discussed here.

We have not included local authority-specific explanatory variables in addition to motor vehicle volume and population size. As far as cycling volume and injuries are concerned, the relationship remains consistent across the cross-sectional and longitudinal models, and is consistent with or without motor vehicle volume coefficients. However, we are not able to draw conclusions about the specific SiN mechanisms at work, for example impacts of infrastructural improvements versus changes in attitudes towards cyclists. Our ability to examine SiN mechanisms is also somewhat limited by the ecological nature of the study and the consequent lack of information about changes in individual behaviour. The longitudinal aspect of the research remains limited with only three time points. Therefore, as with other SiN studies, we cannot be sure that more cycling results in reduced risk, rather than reduced risk resulting in more cycling.

## Conclusion

Our analysis is consistent with the existence of a SiN effect for people cycling in Britain in 1991, 2001 and 2011, and between the years in question. Not only do local authorities with more cycling tend to have a lower per-commuter risk, but places where cycling grew tended to become relatively safer per commuter, and places where cycling declined tended to become relatively less safe per commuter. It is, however, important to look at these findings in context. While some cyclists experienced some benefit from SiN, overall cycling KSIs per commuter rose in Britain during the second half of the period, while KSIs per commuter declined substantially for pedestrians and motor vehicle users. The failure to improve cycling safety during those years is startling by comparison to the improvements made for motor vehicle users and pedestrians, and is a major policy failure.

**What is already known on the subject**

- ‘Safety in Numbers’ (SiN) (more walking or cycling, lower risk per individual walker or cyclist) has been found for walking and cycling.
- However, there are no area-level studies involving time-series analysis that employ robust statistical methods and control for motor traffic volumes.
- The lack of time-series analysis also means that SiN is rarely contextualised in relation to broader changes in safety.

**What this study adds**

- SiN for cyclists in Britain is found using both cross-sectional and time-series data, at a local authority level.
- However, between 2001 and 2011, risk increased per commuter cyclist, showing that SiN is compatible with overall worsening in safety.
- During 2001–2011 risk per commuter declined substantially for motorists and pedestrians, further worsening cycling’s position in relative terms.

10.1136/injuryprev-2017-042498.supp1 Supplementary file 1

10.1136/injuryprev-2017-042498.supp2 Supplementary file 2

## Footnotes

i In 1991, the Journey to Work question was only asked of a 10% sample of those in work, while in 2001 and 2011 all were asked.
