# Supplementary material for: Contextualising Safety in Numbers: a longitudinal investigation into change in cycling safety in Britain, 1991–2001 and 2001–2011
Source: Inj Prev. 2017 Nov 30;25(3):236–41. doi: 10.1136/injuryprev-2017-042498 (PMC6582732; doi:10.1136/injuryprev-2017-042498)
Supplement: Supplementary file 1 [file injuryprev-2017-042498supp001.pdf]

```
clear
clear matrix
* SET TO YOUR WORKING DIRECTORY

use "1b_DataCreated\CensusStats19_LActy.dta", clear

************************
* PREPARE VARIABLES
************************
* FOR CONVENIENCE IN LOOPS, RENAME THE 1993 MEASURE OF MV DISTANCE AS '1991' [1993 used for 1991 time point as nearest available year]
	rename mvkm1993 mvkm1991

* GENERATE FLAG FOR LONDON
	gen lacodesub=substr(lacode,1,3)
	gen london=(lacodesub=="E09")

* GENERATE RELATIVE CHANGE (RATIO) IN NUMBER KSI AND NUMBER CYCLISTS
	foreach x in ncycleksi ncyclemvksi population {
	gen rchange1101_`x'=(`x'2011)/(`x'2001)
	gen rchange0191_`x'=(`x'2001)/(`x'1991)
	}
	foreach x in bicycle mvkm population {
	gen rchange1101_n`x'=(`x'2011)/(`x'2001)
	gen rchange0191_n`x'=(`x'2001)/(`x'1991)
	}

* GENERATE LOGARITHMS
	foreach var of varlist /*
		*/ ncycleksi1991 ncyclemvksi1991 bicycle1991 mvkm1991 population1991 /*
		*/ ncycleksi2001 ncyclemvksi2001 bicycle2001 mvkm2001 population2001 /*
		*/ ncycleksi2011 ncyclemvksi2011 bicycle2011 mvkm2011 population2011 /*
		*/ rchange1101_ncycleksi rchange1101_ncyclemvksi rchange1101_nbicycle rchange1101_nmvkm rchange1101_npopulation /*
		*/ rchange0191_ncycleksi rchange0191_ncyclemvksi rchange0191_nbicycle rchange0191_nmvkm rchange0191_npopulation {
	gen ln`var'=ln(`var')
	}

x

*****************
* ANALYSIS
*****************
** DESCRIPTIVE NATIONAL STATS: TABLE 1
	gen temp=1
	tab temp [fw=commuter2001]
	tab temp [fw=commuter2011]
	total bicycle2001 bicycle2011 foot2001 foot2011
	tab temp [fw=mv2001]
	tab temp [fw=mv2011]
	total nallksi2001 nallksi2011 ncycleksi2001 ncycleksi2011 nfootksi2001 nfootksi2011 nmvksi2001 nmvksi2011


** SiN CROSS SECTIONAL: TABLE 5
	foreach x in 1991 2001 2011 {
	nbreg ncycleksi`x' lnbicycle`x' lnmvkm`x'
```

```
	nbreg ncycleksi`x' lnbicycle`x' lnmvkm`x' lnpopulation`x'
	}

	preserve
	* RESHAPE DATA TO LONG FORMAT
	keep lacode_cty ncycleksi* bicycle* mvkm* population* lnbicycle* lnmvkm* lnpopulation*
	reshape long ncycleksi bicycle mvkm population lnbicycle lnmvkm lnpopulation, i(lacode_cty) j(year)

	* INTERACTION REPORTED IN TEXT: SiN WEAKENS OVER TIME
	gen yearX=year*lnbicycle
	xi: nbreg ncycleksi year lnbicycle yearX lnmvkm
	xi: nbreg ncycleksi year lnbicycle yearX lnmvkm lnpopulation

	* REPEATED MEASURES MODEL IN TABLE 5
	menbreg ncycleksi lnbicycle lnmvkm || lacode_cty:
	menbreg ncycleksi lnbicycle lnmvkm lnpopulation || lacode_cty:

	restore


** SiN LONGITUDINAL: TABLE 6
	glm rchange0191_ncycleksi lnrchange0191_nbicycle lnrchange0191_nmvkm , family(gaussian) link(log)
	glm rchange0191_ncycleksi lnrchange0191_nbicycle lnrchange0191_nmvkm lnrchange0191_npopulation, family(gaussian) link(log)
	glm rchange1101_ncycleksi lnrchange1101_nbicycle lnrchange1101_nmvkm , family(gaussian) link(log)
	glm rchange1101_ncycleksi lnrchange1101_nbicycle lnrchange1101_nmvkm lnrchange1101_npopulation, family(gaussian) link(log)


** SENSITIVITY ANALYSES x2
	* EXTREMELY SIMILAR RESULTS IF CYCLE-MOTOR CRASHES ONLY
		foreach x in 1991 2001 2011 {
		nbreg ncyclemvksi`x' lnbicycle`x' lnmvkm`x'
		nbreg ncyclemvksi`x' lnbicycle`x' lnmvkm`x' lnpopulation`x'
		}
		glm rchange0191_ncyclemvksi lnrchange0191_nbicycle lnrchange0191_nmvkm , family(gaussian) link(log)
		glm rchange0191_ncyclemvksi lnrchange0191_nbicycle lnrchange0191_nmvkm lnrchange0191_npopulation , family(gaussian) link(log)
		glm rchange1101_ncyclemvksi lnrchange1101_nbicycle lnrchange1101_nmvkm , family(gaussian) link(log)
		glm rchange1101_ncyclemvksi lnrchange1101_nbicycle lnrchange1101_nmvkm lnrchange1101_npopulation , family(gaussian) link(log)
	* FAIRLY SIMILAR RESULTS IF EXCLUDE LONDON
		foreach x in 1991 2001 2011 {
		nbreg ncycleksi`x' lnbicycle`x' lnmvkm`x' if london!=1
		nbreg ncycleksi`x' lnbicycle`x' lnmvkm`x' lnpopulation`x' if london!=1
		}
		glm rchange0191_ncycleksi lnrchange0191_nbicycle lnrchange0191_nmvkm if london!=1	, family(gaussian) link(log)
		glm rchange0191_ncycleksi lnrchange0191_nbicycle lnrchange0191_nmvkm lnrchange0191_npopulation if london!=1	, family(gaussian) link(log)
		glm rchange1101_ncycleksi lnrchange1101_nbicycle lnrchange1101_nmvkm if london!=1	, family(gaussian) link(log)
		glm rchange1101_ncycleksi lnrchange1101_nbicycle lnrchange1101_nmvkm lnrchange1101_npopulation if london!=1	, family(gaussian) link(log)
```
