# Supplementary material for: Contextualising Safety in Numbers: a longitudinal investigation into change in cycling safety in Britain, 1991–2001 and 2001–2011
Source: Inj Prev. 2017 Nov 30;25(3):236–41. doi: 10.1136/injuryprev-2017-042498 (PMC6582732; doi:10.1136/injuryprev-2017-042498)
Supplement: Supplementary file 2 [file injuryprev-2017-042498supp002.pdf]

Data sources: DfT (Stats19 data, motor vehicle kilometres data), Nomis/ONS (Census data)

lacode_cty	laname_cty	mvkm1993	mvkm2001	mvkm2011	commuter1	bicycle1991	foot1991	mv1991
E06000001	Hartlepool	551	623	594	31218	900	4692	25270
E06000002	Middlesbro	1134	1233	1143	46266	1160	6760	37907
E06000003	Redcar and	841	889	883	52848	1158	6606	44310
E06000004	Stockton-o	1288	1457	1423	66519	1626	7389	56872
E06000005	Darlington	682	797	781	39540	1140	5852	31908
E06000006	Halton	801	940	973	44907	1322	5631	37202
E06000007	Warrington	2060	2433	2532	81785	3544	7332	70046
E06000008	Blackburn v	615	672	700	50744	474	9085	40550
E06000009	Blackpool	540	574	541	57014	2034	8977	45335
E06000010	Kingston up	1095	1165	1176	91548	12866	9892	67780
E06000011	East Riding	2614	3006	3212	123652	9327	14954	97300
E06000012	North East	837	918	961	63285	5625	7554	49398
E06000013	North Linco	1264	1475	1601	62229	4815	6989	49921
E06000014	York	1013	1185	1217	74354	11740	10524	51123
E06000015	Derby	1538	1599	1689	91243	5062	12365	72923
E06000016	Leicester	1262	1379	1401	102936	3801	17509	81042
E06000017	Rutland	471	530	607	14726	816	2695	10912
E06000018	Nottinghan	1411	1507	1508	95176	2903	13708	77920
E06000019	Herefordsh	1479	1666	1767	65461	3717	8925	52347
E06000020	Telford and	1115	1273	1312	59878	1639	6373	51279
E06000021	Stoke-on-T	1182	1337	1382	100002	1278	17067	81062
E06000022	Bath and N	983	1086	1134	68739	1460	11364	54385
E06000023	Bristol, City	1970	2143	2257	157013	5397	24021	126265
E06000024	North Som	1692	2081	2237	74400	2776	7997	62239
E06000025	South Glou	2738	3379	3747	274652	6212	36681	228769
E06000026	Plymouth	1165	1278	1402	97272	2774	14510	79053
E06000027	Torbay	551	619	671	43409	508	7745	34770
E06000028	Bournemou	732	803	793	58445	2541	5915	49177
E06000029	Poole	730	805	829	57084	2962	5549	48042
E06000030	Swindon	1373	1631	1888	83462	5121	9299	67976
E06000031	Peterborou	1450	1654	1731	65377	7328	6274	50188
E06000032	Luton	709	778	813	73335	1384	9884	57694
E06000033	Southend-c	594	649	654	64593	1735	8046	44634
E06000034	Thurrock	1249	1548	1613	57141	1331	4245	42208
E06000035	Medway	1047	1270	1362	105681	1714	12303	80654
E06000036	Bracknell F	657	720	691	46912	1779	4795	38735
E06000037	West Berks	2286	2856	2923	67849	1939	6741	56606
E06000038	Reading	606	659	525	60823	1783	9342	46827
E06000039	Slough	732	864	859	47576	1796	6185	37137
E06000040	Windsor ar	1552	1829	1817	62706	2043	6762	50268
E06000041	Wokinghan	1487	1662	1652	70071	2482	4670	59268
E06000042	Milton Key	1992	2389	2309	81508	2945	7124	66415
E06000043	Brighton ar	1205	1365	1329	93886	1795	16148	68396
E06000044	Portsmoutl	1135	1214	1218	72709	6611	11451	52403
E06000045	Southampt	1016	1111	1071	81461	3631	9615	66056
E06000046	Isle of Wigl	550	616	621	45326	1759	8611	33992

E06000047	Durham	3111	3627	3817	186055	1420	26815	156302
E06000048	Northumbe	2139	2461	2611	120995	2404	18968	97706
E06000049	Cheshire Ea	3695.296	4366.923	4436	153163	8004	16911	125195
E06000050	Cheshire W	3176.704	3754.077	3849	131903	5607	13544	110724
E06000051	Shropshire	2440	2813	2927	114843	5331	17257	91055
E06000052	Cornwall,Is	3516	4137	4749	166608	3380	27580	133460
E06000054	Wiltshire	4402	5065	5437	179834	8733	28466	139419
E06000055	Bedford	964.7158	1106.594	1150	60346	3829	6825	47168
E06000056	Central Bec	2360.284	2707.406	2850	105246	3729	10774	84930
E08000001	Bolton	1761	2029	2039	104147	1398	13168	87324
E08000002	Bury	1285	1486	1617	77645	1113	8506	65025
E08000003	Mancheste	2468	2700	2615	129972	4450	17706	104958
E08000004	Oldham	808	1040	1055	88031	1181	12602	73025
E08000005	Rochdale	1382	1660	1737	80545	977	11699	66629
E08000006	Salford	2036	2268	2311	81233	2007	11304	66808
E08000007	Stockport	1583	1847	1809	127178	3176	12726	105861
E08000008	Tameside	832	1086	1149	92037	1451	12338	75987
E08000009	Trafford	1431	1518	1446	89855	3498	8185	74656
E08000010	Wigan	1620	1868	1909	124560	2402	15172	105142
E08000011	Knowsley	1114	1273	1384	46954	876	5731	37899
E08000012	Liverpool	2025	2158	2152	140179	2292	16992	115715
E08000013	St. Helens	1076	1225	1311	69497	931	8271	58809
E08000014	Sefton	1053	1125	1121	108912	4069	10822	84387
E08000015	Wirral	1412	1593	1670	123052	2535	12828	98458
E08000016	Barnsley	1472	1761	1852	80807	633	11976	67257
E08000017	Doncaster	2276	2739	2959	102617	4810	12999	83145
E08000018	Rotherham	1892	2193	2305	97544	1119	10706	84884
E08000019	Sheffield	2290	2622	2643	199037	1693	21293	174521
E08000020	Gateshead	1680	1814	1705	76298	494	8760	61537
E08000021	Newcastle	1669	1798	1697	92908	1352	11988	73681
E08000022	North Tyne	1172	1274	1251	74990	1697	8940	56216
E08000023	South Tyne	687	749	721	52966	987	6758	39411
E08000024	Sunderlanc	1561	1734	1685	101139	1040	12778	84883
E08000025	Birminghar	5608	5889	5786	350923	4845	36917	299738
E08000026	Coventry	1604	1756	1801	114369	3702	13968	95155
E08000027	Dudley	1557	1707	1656	135456	1777	15964	115297
E08000028	Sandwell	1758	1927	1955	112206	1892	17389	91321
E08000029	Solihull	1867	2110	2260	90408	1581	5921	79309
E08000030	Walsall	1322	1493	1464	103786	2598	13212	87103
E08000031	Wolverhan	1118	1179	1165	89192	2888	11121	73341
E08000032	Bradford	2137	2338	2394	176579	1462	23404	147710
E08000033	Calderdale	1285	1462	1486	82092	710	10846	69111
E08000034	Kirklees	2185	2537	2596	155013	1328	20045	131353
E08000035	Leeds	5225	5945	6112	286743	3517	35462	243139
E08000036	Wakefield	2138	2530	2754	126312	1767	19059	103378
E09000002	Barking anc	552	560	584	55524	1064	6200	33589
E09000003	Barnet	1548	1645	1587	122473	1000	9328	79821
E09000004	Bexley	870	981	902	96653	960	6731	62796
E09000005	Brent	909	965	873	100038	1261	9526	58044
E09000006	Bromley	1264	1346	1200	130108	1374	9639	82995

E09000007	Camden	619	605	466	68636	2099	13122	30974
E09000008	Croydon	1321	1350	1153	143263	1541	14642	98036
E09000009	Ealing	1265	1376	1215	120301	2354	10440	76090
E09000010	Enfield	1436	1522	1479	110916	2028	10171	75892
E09000011	Greenwich	1081	1128	1079	80826	973	8211	48567
E09000012	Hackney	527	555	482	58461	2507	7246	35818
E09000013	Hammersm	579	600	531	65725	2664	8686	28435
E09000014	Haringey	618	619	539	78543	1566	5993	42352
E09000015	Harrow	592	628	569	89773	930	6928	59194
E09000016	Havering	1360	1507	1480	102733	1053	7761	65545
E09000017	Hillingdon	2051	2238	2036	109298	3055	9981	81588
E09000018	Hounslow	1591	1662	1499	92288	3692	8604	63439
E09000019	Islington	485	479	409	63059	2379	10237	31848
E09000020	Kensington	570	584	520	57723	1621	8688	25635
E09000021	Kingston u	983	1002	901	62982	2109	6602	42153
E09000022	Lambeth	909	924	746	96509	3131	9527	46741
E09000023	Lewisham	850	883	772	94421	1626	7940	52032
E09000024	Merton	655	683	584	76154	1803	6329	43594
E09000025	Newham	857	893	904	69304	1005	7250	35608
E09000026	Redbridge	943	1053	1106	96629	1223	6439	56362
E09000027	Richmond u	884	902	766	75353	3467	6204	48064
E09000028	Southwark	850	877	751	79980	2437	10900	47321
E09000029	Sutton	697	722	612	78438	1663	7655	55160
E09000030	Tower Ham	895	947	904	48051	1232	9622	22929
E09000031	Waltham F	709	771	651	87471	1418	8372	48373
E09000032	Wandswor	990	1016	795	115928	3751	11917	56736
E09000033	Westminst	1256	1237	1008	74973	1785	18398	31934
E10000002	Buckinghar	5082	6194	6001	215237	4713	21132	178799
E10000003	Cambridge:	5672	6744	7154	227768	25252	21595	175113
E10000006	Cumbria	4565	5217	5368	205270	6991	38146	158021
E10000007	Derbyshire	6079	7041	7561	306377	6961	42686	252501
E10000008	Devon	6011	7065	7763	252809	6580	41224	202143
E10000009	Dorset	3111	3576	3763	143211	5637	18984	116727
E10000011	East Sussex	3633	4084	4125	178334	3450	23687	140679
E10000012	Essex	11284	12962	13653	549743	18432	52507	406126
E10000013	Gloucester	4658	5673	6185	235514	13009	28136	192408
E10000014	Hampshire	12161	14358	14689	543267	25289	57182	441028
E10000015	Hertfordsh	9619	11004	11366	456741	11307	49355	349500
E10000016	Kent	11382	13682	14344	538020	14346	69449	406479
E10000017	Lancashire	9181	10379	10953	458748	13296	63952	375770
E10000018	Leicestersh	5566	6913	7219	265489	10313	32402	220278
E10000019	Lincolnshir	4487	5264	5830	237497	18167	31695	185088
E10000020	Norfolk	6656	7595	7954	307006	22472	36135	244851
E10000021	Northampt	6421	7707	8116	266631	7253	33425	221570
E10000023	North York	6250	7208	7813	227745	8918	39130	176876
E10000024	Nottinghar	5422	6222	6845	311162	11778	37328	259839
E10000025	Oxfordshir	6059	7143	7207	257168	21901	30047	199027
E10000027	Somerset	4761	5609	6202	192060	11637	27417	151427
E10000028	Staffordshi	7269	8347	9084	354397	8817	39966	301027
E10000029	Suffolk	4888	5672	5793	277603	18456	33383	221350

E10000030	Surrey	11664	13756	13508	473502	14605	41308	368431
E10000031	Warwicksh	6859	8195	8733	218096	8561	26417	180114
E10000032	West Susse	6299	7320	7051	300005	13904	34734	232348
E10000034	Worcesters	5366	6322	6535	231509	7195	27178	193825
S12000005	Clackmann	243	273	311	19052	245	2866	15744
S12000006	Dumfries a	1561	1817	1958	57113	1531	11681	43499
S12000008	East Ayrshi	779	931	1021	44743	141	6815	37197
S12000009	East Dunba	471	513	529	48026	211	3651	40761
S12000010	East Lothia	653	765	847	34760	538	4570	28810
S12000011	East Renfre	508	590	750	36467	151	2422	31161
S12000013	Eilean Siar	156	176	200	9614	41	1333	8098
S12000014	Falkirk	1114	1326	1477	58240	1145	7582	47347
S12000015	Fife	2267	2554	2816	137582	2277	20754	110577
S12000017	Highland	2036	2324	2559	81662	3140	14180	63008
S12000018	Inverclyde	463	516	511	31755	40	4569	25687
S12000019	Midlothian	520	603	647	34634	244	4106	30141
S12000020	Moray	571	655	700	33687	2163	6914	23645
S12000021	North Ayrsl	634	671	762	50126	549	6904	40769
S12000023	Orkney Isla	263	302	331	17084	243	2800	13710
S12000024	Perth and K	1860	2146	2246	53786	1029	9746	42431
S12000026	Scottish Bo	967	1075	1175	44105	767	12116	30988
S12000028	South Ayrsl	773	890	968	43745	649	6243	35633
S12000029	South Lana	1825	2106	2427	121696	457	15093	99844
S12000030	Stirling	944	1111	1204	31495	283	4440	25712
S12000033	Aberdeen C	1160	1293	1281	97635	1768	14248	79845
S12000034	Aberdeensl	2237	2470	2657	92003	1678	14149	73476
S12000035	Argyll and E	719	796	874	35729	508	8014	25855
S12000036	Edinburgh	2476	2801	2866	185925	3520	27913	152253
S12000038	Renfrewshi	1174	1313	1449	71949	512	8709	58741
S12000039	West Dunb	521	583	633	36394	223	5159	27979
S12000040	West Lothi	1293	1562	1706	64998	392	7201	55121
S12000041	Angus	823	915	1057	44950	1632	8464	34001
S12000042	Dundee Cit	761	813	856	57087	615	8747	47138
S12000043	Glasgow Ci	2676	3104	3323	199359	1624	27540	153690
S12000044	North Lana	2491	2834	2943	121070	314	15612	99878
W0600000	Isle of Angl	426	560	595	22938	567	3220	18919
W0600000	Gwynedd	1029	1139	1248	39888	628	6498	32424
W0600000	Conwy	892	990	1091	38783	646	5689	31998
W0600000	Denbighshi	694	772	858	31285	708	4716	25585
W0600000	Flintshire	1374	1529	1591	60549	1516	6583	51951
W0600000	Wrexham	768	872	919	47685	779	5746	40897
W0600000	Ceredigion	599	657	695	20783	180	2830	17695
W0600000	Pembrokes	841	961	1053	39746	334	6022	33089
W0600001	Carmarther	1460	1621	1838	57266	602	7550	48600
W0600001	Swansea	1350	1534	1632	79668	738	8814	69445
W0600001	Neath Port	955	1142	1296	47268	849	6263	39552
W0600001	Bridgend	964	1160	1252	48723	484	5430	42479
W0600001	The Vale of	892	980	982	46765	1009	5335	38097
W0600001	Cardiff	2453	2813	2768	110609	2951	14746	90276
W0600001	Rhondda, C	1670	1874	2025	80194	401	10477	67094

W0600001	Caerphilly,	1248	1382	1510	40572	276	5720	34330
W0600001	Blaenau Gv	328	356	397	23314	70	4151	19009
W0600002	Torfaen	495	557	603	34011	407	5035	28163
W0600002	Monmouth	1041	1225	1339	32877	685	4038	27694
W0600002	Newport	1421	1712	1787	50752	758	6230	42972
W0600002	Powys	1192	1318	1453	45892	816	8336	36345

lacode_cty	Local authority code
laname_cty	Local authority name
mvkm1993	Motor vehicle kilometres per county
mvkm2001	Motor vehicle kilometres per county
mvkm2011	Motor vehicle kilometres per county
commuter1	Commuters
bicycle1991	Bicycle commuters
foot1991	Pedestrian commuters
mv1991	Motor vehicle commuters
population	Population
commuter2	Commuters
bicycle2001	Bicycle commuters
foot2001	Pedestrian commuters
mv2001	Motor vehicle commuters
population	Population
commuter2	Commuters
bicycle2011	Bicycle commuters
foot2011	Pedestrian commuters
mv2011	Motor vehicle commuters
population	Population
nallksi1991	All KSIs
nallksi2001	All KSIs
nallksi2011	All KSIs
ncycleksi19	Cycle KSIs
ncycleksi20	Cycle KSIs
ncycleksi20	Cycle KSIs
ncyclemvks	Cycle KSIs (not involving motor vehicles)
ncyclemvks	Cycle KSIs (not involving motor vehicles)
ncyclemvks	Cycle KSIs (not involving motor vehicles)
nfootksi19	Pedestrian KSIs
nfootksi20	Pedestrian KSIs
nfootksi20	Pedestrian KSIs
nmvksi199	Motor vehicle user KSIs
nmvksi200	Motor vehicle user KSIs
nmvksi201	Motor vehicle user KSIs

population	commuter2	bicycle200	foot2001	mv2001	population	commuter2	bicycle201	foot2011
0.090409	31563	937	3935	26140	0.088611	35294	706	4305
0.140849	46132	1251	5791	38320	0.134855	51210	1375	6769
0.145123	50397	1052	5404	42708	0.139132	52320	875	5335
0.173912	70455	1576	6302	61393	0.178408	80746	1693	7101
0.098906	39440	949	5085	32638	0.097838	44834	1151	6284
0.123716	47263	1023	4923	40450	0.118208	53881	1191	5221
0.182685	83745	2936	6599	72602	0.19108	91520	2577	7038
0.136612	49220	622	7062	40808	0.13747	55593	592	7460
0.146069	52992	1885	8287	41746	0.142283	55246	1916	8705
0.254117	90761	11181	9960	68400	0.243589	105227	8944	12705
0.291977	129938	7420	13967	105639	0.314113	139815	5819	14774
0.159662	61513	5038	6569	49062	0.157979	66202	3817	7841
0.152284	62424	3793	6289	51676	0.152849	71260	2959	7041
0.166765	80434	10508	13049	55148	0.181094	89424	11106	17457
0.218802	88938	4233	11413	72182	0.221708	104593	4158	13755
0.270493	103183	4462	17660	79641	0.279921	128102	4980	22303
0.031489	14400	811	2279	10949	0.034563	15026	606	2478
0.263522	94912	3731	14980	75033	0.266988	114362	4257	19064
0.160183	68910	3415	9479	55093	0.174871	75114	3223	11062
0.139516	68611	1864	6147	59597	0.158325	72041	1644	5840
0.244637	92282	1543	11563	78463	0.240636	101176	1574	10739
0.158692	71999	1949	12095	55578	0.16904	74234	2451	13636
0.376146	164113	8108	27607	126147	0.380615	192154	15768	38832
0.177472	79076	2260	7722	67387	0.188564	85829	2646	8460
0.686741	116848	3782	9339	102438	0.245641	123744	5284	10187
0.243373	99287	2806	14035	81138	0.24072	109568	3055	16592
0.119674	47736	831	8371	37863	0.129706	50059	779	8413
0.151302	65407	2892	7911	53170	0.163444	80075	4169	10919
0.13305	58534	2791	5661	48985	0.138288	64258	3393	6859
0.17085	87296	4777	9712	71612	0.180051	100650	4592	10395
0.153166	67771	5643	6256	53969	0.156061	80886	4990	7506
0.171671	76335	1432	9564	60879	0.184371	83030	1149	11261
0.158517	64560	1917	8002	44978	0.160257	73905	2260	10629
0.127819	64733	1165	4341	49047	0.143128	72234	1078	4230
0.240228	110176	1620	11381	87103	0.249488	116767	1419	11643
0.095949	54579	1672	5240	45210	0.109617	55039	1360	4602
0.1367	69067	2071	6712	56817	0.144483	70990	2064	6644
0.128877	68148	3028	11306	48717	0.143096	72853	3252	13087
0.105625	54134	1714	6035	43156	0.119067	62323	1566	6169
0.132465	60344	1908	6250	47253	0.133626	63053	1684	6131
0.139189	72604	2153	4878	60939	0.150229	70535	1943	4456
0.17633	98822	3265	7405	83717	0.207057	115295	3599	9117
0.228946	106681	3168	20162	72681	0.247817	122467	6635	27242
0.174697	81147	6160	12894	59318	0.186701	89527	7008	15568
0.196864	91699	4226	13063	71861	0.217445	104841	5138	17826
0.124577	48687	1658	9081	36066	0.132731	52019	1771	9463

0.494524	186662	1888	21178	161102	0.49347	207242	2205	21490
0.304694	121396	2176	15519	100679	0.30719	129007	2074	14632
0.339279	150721	5767	15270	125568	0.351817	157799	4666	16282
0.310936	135676	4465	12993	115377	0.321971	142960	4200	14669
0.266871	117354	4701	17114	93589	0.283173	127940	4310	16951
0.470473	184351	3770	27926	148973	0.501267	206814	3963	29293
0.393621	193093	7885	26396	153632	0.432973	206515	6638	26680
0.133692	63844	3222	6796	50681	0.147911	67889	2866	7360
0.218742	109288	2726	9553	90639	0.233661	117280	2058	9554
0.258584	105350	1413	11587	89050	0.261037	113281	1261	11435
0.17676	77113	1047	6847	64873	0.180608	80347	1176	7094
0.404861	133131	4610	16163	108030	0.392819	198417	8426	29347
0.216531	85560	1006	10115	72619	0.217273	88951	993	9622
0.202164	80139	1043	9323	68007	0.205357	83677	910	8172
0.220463	83290	2042	10134	68790	0.216103	98917	2491	13850
0.284395	124092	2540	10953	105233	0.284528	123768	2756	10637
0.216431	88810	1507	10080	74681	0.213043	94241	1401	9519
0.212731	90537	2988	7624	73640	0.210145	99746	3393	8338
0.306521	125905	2386	13073	107963	0.301415	139044	2354	13301
0.152091	51385	904	5037	42878	0.150459	58160	962	5569
0.45245	146117	2686	16393	121033	0.439473	184450	3970	23991
0.178764	68271	1152	6331	58901	0.176843	73233	1092	6231
0.289542	107581	3365	11096	85391	0.282958	111782	3176	11561
0.330795	117418	2298	10497	96349	0.312293	128327	2275	11193
0.220937	82185	734	10186	69914	0.218063	95634	712	9700
0.288854	109142	3420	11497	91910	0.286866	122760	3227	12214
0.251637	99098	1007	8751	87791	0.248175	106123	987	8542
0.501202	203180	2365	22774	169549	0.513234	225765	4267	29288
0.199588	73304	816	6972	60940	0.191151	85494	1314	7966
0.259541	94432	1781	11235	74326	0.259536	110905	3223	15302
0.192286	77645	1692	7092	60016	0.191659	89029	2345	7559
0.154697	55156	1143	5543	42319	0.152785	60747	1396	5749
0.28904	107078	1525	11215	91326	0.280807	113806	1588	11310
0.961041	339693	5133	32265	291101	0.977087	390854	6458	40042
0.294387	117543	3573	13391	98402	0.300848	127517	3594	15042
0.304615	129513	1872	12101	112625	0.305155	131042	1723	10645
0.290091	105791	1972	11658	88974	0.282904	120790	1996	10936
0.199859	84851	1446	5312	74372	0.199517	87952	1341	5516
0.259488	97650	2155	10587	83570	0.253499	104120	1747	9460
0.24219	87842	2534	9579	72712	0.236582	97055	2170	9870
0.457344	175577	1481	20686	146390	0.467665	199625	1681	23175
0.191585	80335	709	9487	67935	0.192405	87139	877	9763
0.373127	159342	1765	17570	136166	0.388567	174681	1791	17802
0.680722	298857	4189	33844	254643	0.715402	325578	6237	40140
0.310915	128477	2001	14853	108557	0.315172	140455	1869	14373
0.143681	61854	1021	4665	38342	0.163944	70477	1064	4420
0.293564	130453	1351	8772	78760	0.314564	149690	2368	9551
0.215615	96092	1039	5878	63258	0.218307	101599	1274	5712
0.243025	107818	1933	7614	59741	0.263464	134605	3699	9877
0.290609	128844	1333	8792	76407	0.295532	135569	2190	8708

0.170444	82008	3362	14654	28464	0.19802	94600	6721	16420
0.31351	143369	1615	12705	88964	0.330587	157368	2115	12296
0.275257	131510	3157	9748	77577	0.300948	149818	4760	10974
0.257417	111272	1437	7839	73611	0.273559	125260	1905	8268
0.20765	85005	1336	6295	47919	0.214403	108211	2669	7110
0.181248	72289	4940	7811	39603	0.202824	106593	16389	13187
0.148502	75647	3942	10068	26918	0.165242	89202	7103	11980
0.202204	87316	2388	5657	39695	0.216507	111831	5911	7199
0.2001	88115	848	6180	56569	0.206814	102326	869	6746
0.229492	97037	914	6407	62772	0.224248	104141	998	6584
0.231602	107420	2019	7933	82183	0.243006	119061	1892	8300
0.204397	94956	3185	8265	64313	0.212341	115893	4050	8783
0.164686	73144	3770	11972	30780	0.175797	96643	9763	16059
0.138394	65312	2130	9529	24536	0.158919	67877	3701	9148
0.132996	68110	2331	7207	41750	0.147273	73015	3278	7697
0.244834	120863	5407	9250	48307	0.266169	152632	12930	11898
0.230983	106233	2121	7342	53267	0.248922	124644	5164	8227
0.16847	86891	2220	6485	42756	0.187908	95367	3409	6845
0.21217	79375	1188	6351	36918	0.243891	123509	2163	8554
0.226218	97147	966	6115	55184	0.238635	113663	1356	6722
0.160732	79556	3493	6881	44472	0.172335	85334	5849	7202
0.218541	99555	3965	12202	49767	0.244866	135085	10450	17426
0.16888	82880	1930	7260	56115	0.179768	88920	2019	7184
0.161064	68280	2213	11667	22555	0.196106	111827	7785	20672
0.212033	91042	1708	6915	45920	0.218341	111828	3306	7500
0.252425	130382	5498	10863	48423	0.26038	161947	12804	12494
0.178956	83424	2570	20018	27157	0.188471	99697	5335	22123
0.454692	213317	3890	20315	174563	0.479026	219000	3389	19348
0.491959	247944	24956	22320	192234	0.552658	278569	29689	26148
0.483163	193512	5931	31558	152879	0.487607	212055	5945	33445
0.709834	309964	6199	34565	263265	0.734585	333436	5498	32704
0.646903	269559	7276	43302	213782	0.704493	297142	9140	47330
0.360814	151088	5259	19396	123568	0.39098	162881	5302	20557
0.461501	186731	3684	22935	147724	0.492324	206124	3826	24822
1.242241	567553	15687	51297	423992	1.310835	604911	13891	56356
0.52837	243725	11417	30060	199154	0.564559	262734	11090	32195
1.169986	564981	22092	54523	463837	1.240103	588535	20658	54154
0.975829	470002	9245	45744	358883	1.033977	502593	9175	48180
1.268645	549743	12210	64317	424071	1.329718	611561	11622	70023
1.101317	459718	12345	55775	383469	1.134974	492366	11359	55041
0.565539	274693	9471	27537	233855	0.609578	288978	8496	26910
0.584536	260491	15789	31860	208856	0.646645	295051	13092	35868
0.745613	321613	18668	38257	258798	0.796728	351908	17332	41383
0.578807	286562	7089	29979	244209	0.629676	313537	6512	31040
0.535396	237395	7773	39009	184708	0.56966	253584	6572	39691
0.73035	311277	10859	30974	265003	0.74851	337683	10090	31715
0.547584	275605	20674	31441	214866	0.605488	292694	23101	36630
0.460368	202587	10627	28765	160739	0.498093	219938	9642	31556
0.786498	350545	8359	33937	302384	0.806744	369612	7253	33739
0.636266	282250	15532	31607	228323	0.668553	311418	13558	36995

1.014909	475676	11900	42477	362219	1.059015	498073	12440	42817
0.484247	225379	7313	24213	189727	0.50586	240932	6557	25056
0.70229	320313	12222	35035	250300	0.753614	346443	11681	38155
0.516564	239780	6665	25001	202827	0.542107	244720	5653	24698
0.047679	18811	222	1916	16344	0.048077	20763	208	1678
0.147805	56180	1350	10353	43458	0.147765	57364	1015	8710
0.122455	46147	230	5286	39482	0.120235	48137	219	4014
0.1094	45626	315	2597	38913	0.108243	42910	419	2264
0.084114	38225	492	3878	32043	0.090088	41360	617	3524
0.083635	37018	225	1738	31757	0.089311	35960	314	1413
0.0296	10084	95	1103	8411	0.026502	10978	72	948
0.14098	61519	943	5514	52145	0.145191	67561	738	4737
0.341199	142025	2034	15608	118445	0.349429	145508	1733	12689
0.204004	85648	2748	14330	65706	0.208914	94822	2786	12972
0.090103	31906	90	3290	26969	0.084203	30686	91	2831
0.078845	36188	341	3176	32400	0.080941	35892	384	2538
0.083616	37109	2102	6019	27161	0.08694	39379	1140	5466
0.136875	50344	626	5189	41622	0.135817	50806	418	4264
0.042134	18127	259	2839	14455	0.041233	19831	205	2555
0.128044	55565	702	9372	44195	0.134949	59734	810	8831
0.103881	44179	771	9287	33403	0.106764	44804	561	7119
0.112658	43031	656	4762	35669	0.112097	42422	601	4098
0.297202	123195	521	10839	105714	0.302216	131285	574	9594
0.078833	33800	411	4024	27701	0.086212	33961	446	3581
0.204885	94655	1662	14365	75334	0.212125	101126	2018	16962
0.215387	100361	1487	11969	82408	0.226871	114535	1050	10121
0.091623	36581	574	7873	25917	0.091306	33545	503	5930
0.418914	193798	6072	33164	150013	0.448624	197878	9478	36059
0.173304	72785	654	5751	61540	0.172867	71648	624	4977
0.096407	36313	252	3657	28768	0.093378	36099	257	3040
0.144137	72189	598	5673	62845	0.158714	76223	607	5155
0.10881	44970	1161	7242	34955	0.1084	47091	783	5756
0.146994	51941	656	7494	43017	0.145663	52901	733	7709
0.611218	193700	2234	24605	147151	0.577869	217354	3963	29124
0.323826	127561	371	10493	109770	0.321067	138801	454	9604
0.069149	22907	448	2610	19444	0.066829	26405	453	2465
0.113331	39927	741	6566	32037	0.116843	45415	677	6724
0.10632	38126	740	4742	31932	0.109596	43350	645	4954
0.088213	33851	697	4390	28151	0.093065	36020	633	4395
0.141344	64037	1189	4988	57074	0.148594	67472	1294	5079
0.123354	52231	908	4989	45826	0.128476	58290	808	5128
0.063094	23864	309	3837	19401	0.074941	25666	361	4266
0.112085	37520	364	5190	31349	0.114131	44822	435	5106
0.168364	57816	716	6099	50024	0.172842	69050	615	6369
0.223192	81456	1024	8232	71171	0.223301	94565	1485	9997
0.138236	46750	674	4794	40473	0.134468	54899	755	4539
0.129249	49163	502	4972	42805	0.128645	56446	503	4967
0.116918	47709	980	4364	40001	0.119292	53092	998	4701
0.285402	121579	3514	17196	97670	0.305353	147616	5791	23644
0.232595	82773	449	8561	71069	0.231946	92549	441	7836

0.122411	81569	577	8133	70527	0.2255	95560	555	7802
0.072254	23688	146	3135	20244	0.070064	26631	134	2574
0.090527	34585	378	3483	30325	0.090949	37679	326	3089
0.079936	33365	474	3823	28355	0.084885	36198	483	3792
0.133318	52514	820	5249	45433	0.137011	59680	921	5652
0.119298	45503	739	7379	36763	0.126354	50032	843	7097

mv2011	population	nallksi1991	nallksi2001	nallksi2011	ncycleksi19	ncycleksi20	ncycleksi20	ncyclemvks
29424	0.092028	193	122	88	18	9	15	18
41759	0.138412	307	237	97	22	15	14	21
44214	0.135177	262	168	114	19	25	9	19
69833	0.19161	359	223	175	38	20	31	35
36292	0.105564	226	133	104	25	7	14	25
46087	0.125746	201	266	121	14	24	16	14
79311	0.202228	335	466	321	34	33	50	33
46268	0.147489	389	242	199	20	11	11	20
43684	0.142065	466	313	191	31	25	31	26
82084	0.256406	775	439	363	146	83	91	124
115776	0.334179	1091	893	565	95	57	49	78
53628	0.159616	506	382	237	69	65	38	57
60469	0.167446	615	439	303	64	60	36	56
57949	0.198051	356	361	176	77	45	43	65
84913	0.248752	387	368	264	43	52	53	40
98303	0.329839	593	272	270	65	23	53	62
11540	0.037369	128	96	71	4	4	2	4
86538	0.30568	1116	746	425	120	87	88	102
59586	0.183477	541	501	216	68	34	17	56
63121	0.166641	447	264	116	36	12	13	34
87622	0.249008	610	314	161	48	20	16	44
54764	0.176016	372	207	94	21	10	15	19
132469	0.428234	790	596	435	60	68	121	58
71995	0.202566	415	296	160	36	11	19	35
106109	0.262767	522	378	169	38	23	25	37
88203	0.256384	922	303	195	95	30	24	94
39993	0.130959	192	128	101	9	5	7	8
62937	0.183491	342	305	258	41	38	58	41
52451	0.147645	315	159	177	36	15	40	34
83801	0.209156	460	321	213	62	28	41	59
65788	0.183631	698	422	255	133	46	45	103
64603	0.203201	294	192	166	26	14	18	25
47890	0.173658	370	293	191	47	39	28	41
53566	0.157705	430	338	205	20	19	18	16
91952	0.263925	484	313	174	38	12	20	33
45782	0.113205	163	162	84	20	9	9	19
58089	0.153822	463	385	216	27	19	24	23
48554	0.155698	229	216	131	24	24	34	22
49024	0.140205	198	238	129	18	16	20	17
48123	0.14456	289	295	167	34	20	23	31
58117	0.15438	238	247	121	20	15	10	18
96502	0.248821	352	477	266	29	25	25	28
73684	0.273369	548	477	468	38	38	93	36
62733	0.205056	501	365	357	95	52	94	86
77913	0.236882	587	339	387	69	48	97	57
38676	0.138265	377	355	267	38	28	23	29

180177	0.513242	1085	685	577	49	45	30	46
108647	0.316028	1031	645	478	45	36	34	34
130864	0.370127	919	971	732	81	51	100	57
119926	0.329608	792	704	627	57	44	78	51
104140	0.306129	1326	928	392	84	42	35	65
169157	0.534476	1619	994	634	91	37	41	84
165925	0.470981	1596	1043	711	92	49	53	83
53790	0.157479	378	264	171	36	17	33	32
96801	0.254381	774	519	342	44	14	26	40
95399	0.276786	678	328	241	35	21	30	31
66996	0.18506	339	165	153	25	10	23	24
151002	0.503127	1259	826	535	100	71	112	99
76592	0.224897	470	240	204	39	14	14	36
72192	0.211699	473	271	145	33	20	9	31
77920	0.233933	594	296	210	45	27	31	44
102851	0.283275	452	252	171	53	28	32	50
79298	0.219324	522	261	162	39	23	15	38
80518	0.226578	337	218	146	35	25	31	32
119488	0.317849	643	406	218	44	32	28	41
47675	0.145893	407	190	160	32	15	21	31
144538	0.466415	1376	978	667	94	52	96	80
63225	0.175308	409	253	188	29	12	18	27
86866	0.27379	439	313	250	41	27	41	41
104142	0.319783	661	508	333	60	44	52	54
82697	0.231221	508	426	243	26	20	13	24
103869	0.302402	696	448	409	44	36	58	44
94544	0.25728	600	346	230	42	18	18	40
178919	0.552698	952	921	503	46	64	67	43
70792	0.200214	551	306	227	29	16	26	27
83981	0.280177	701	329	268	42	29	39	41
68319	0.200801	398	198	140	25	19	18	24
45623	0.148127	291	126	103	26	16	15	25
96374	0.275506	820	356	260	55	35	33	52
321838	1.073045	2686	1779	1283	143	79	140	141
105194	0.31696	1233	548	342	121	48	43	121
113973	0.312925	660	374	283	35	34	31	35
102172	0.308063	767	581	333	56	41	36	55
75482	0.206674	669	317	188	43	21	17	42
90606	0.269323	627	373	205	47	26	19	47
80207	0.24947	724	360	254	66	27	36	66
162528	0.522452	1315	892	645	72	51	59	57
72927	0.203826	513	337	267	32	21	31	27
148918	0.422458	1001	704	448	59	45	50	45
266075	0.751485	2127	1451	904	114	89	122	109
119627	0.325837	1168	564	468	79	27	51	77
39610	0.185911	468	325	144	31	14	18	30
83994	0.356386	1066	767	385	63	19	38	62
64381	0.231997	533	392	172	34	19	22	31
68832	0.311215	991	581	242	49	26	24	48
73807	0.309392	856	608	261	61	22	39	57

28066	0.220338	803	705	326	71	80	86	68
89766	0.363378	1007	756	303	65	25	40	62
79639	0.338449	915	615	239	58	50	30	57
77809	0.312466	551	693	282	37	21	22	36
53099	0.254557	814	575	271	42	30	27	39
45575	0.24627	583	604	358	42	61	119	39
27591	0.182493	526	414	231	57	51	67	56
45609	0.254926	385	583	264	17	25	35	15
58881	0.239056	565	311	122	55	15	9	53
65644	0.237232	792	494	215	34	21	13	31
86038	0.273936	815	587	252	77	30	23	76
73113	0.253957	891	579	243	80	38	33	75
33360	0.206125	500	618	303	48	103	107	41
23203	0.158649	538	486	256	57	71	71	52
40705	0.16006	481	261	124	59	25	33	54
54722	0.303086	1165	812	476	103	80	120	98
53741	0.275885	894	648	312	57	34	57	48
41105	0.199693	502	317	150	47	33	27	45
45224	0.307984	700	435	232	38	28	33	37
57595	0.27897	569	521	245	40	24	16	39
40719	0.18699	476	297	193	73	31	61	65
58409	0.288283	1144	717	408	102	76	107	95
56747	0.190146	560	337	136	45	23	20	44
29409	0.254096	650	475	362	44	30	105	44
49386	0.258249	598	538	204	35	26	38	34
52096	0.306995	899	570	323	86	59	83	81
29478	0.226771	1821	1257	687	150	110	189	144
176804	0.505283	1200	1226	651	76	61	52	66
209538	0.62121	2577	1602	973	409	161	172	303
168717	0.499858	1952	1455	678	90	78	56	73
287016	0.769686	1573	1810	982	81	93	96	71
233195	0.746399	2309	1258	834	126	75	75	113
133350	0.412905	1134	1031	636	70	64	73	66
160240	0.526671	1392	1158	925	62	49	79	58
446783	1.393587	3581	3319	1894	311	207	160	256
214696	0.596984	1614	1115	686	111	88	104	109
481388	1.317788	4164	2799	2080	364	202	281	326
367285	1.116062	3359	2605	1177	273	162	135	246
463924	1.46374	4018	3077	1588	293	171	154	247
414853	1.171339	3092	2755	1901	212	231	222	193
248595	0.650489	1542	1112	671	102	42	37	83
241050	0.713653	2534	1941	1373	162	78	110	151
285414	0.857888	3623	2044	1060	300	145	96	251
268517	0.691952	2733	1732	950	124	104	84	123
199259	0.598376	4067	2470	1418	223	99	121	171
288408	0.785802	2757	2154	1313	270	147	154	234
221668	0.653798	1773	1538	1057	162	120	196	149
175390	0.529972	1532	1010	674	106	48	72	98
319775	0.848489	1943	1123	636	113	50	50	97
251577	0.728163	2436	1395	984	197	104	117	170

361853	1.13239	3420	2085	1676	310	148	329	245
202423	0.545474	2537	1840	913	197	91	81	175
265746	0.806892	2011	1631	1248	182	137	153	170
206927	0.566169	1578	1014	490	129	65	52	105
18112	0.051442	138	120	52	8	6	8	8
46714	0.151324	726	421	254	31	17	9	26
42535	0.122767	522	279	148	21	8	9	20
35809	0.105026	242	126	68	11	5	6	10
34357	0.099717	227	176	90	7	2	11	7
30423	0.090574	269	125	54	9	3	12	7
9299	0.027684	96	58	25	3	1	1	1
57654	0.15599	474	272	162	37	15	15	31
123878	0.365198	820	759	342	47	28	22	37
75449	0.232132	1283	838	350	60	30	13	49
25500	0.081485	263	120	75	9	8	3	9
32582	0.083187	213	164	86	13	9	6	10
30251	0.093295	247	191	112	15	8	3	13
42316	0.138146	550	240	111	28	8	5	27
16280	0.044516	126	67	32	2	3	2	2
48614	0.146652	906	568	307	35	12	18	26
36471	0.11387	462	342	244	15	7	18	11
35687	0.112799	517	286	133	25	10	8	21
111690	0.31383	1045	617	266	32	18	15	28
27909	0.090247	384	325	183	24	12	11	16
78572	0.222793	498	217	302	33	12	23	27
97525	0.252973	931	581	645	38	15	15	30
25026	0.088166	595	389	211	17	19	4	15
146392	0.476626	1219	761	511	105	79	108	85
60806	0.174908	651	344	177	25	24	24	22
29166	0.09072	282	159	73	17	9	7	14
66075	0.175118	349	255	189	12	12	17	10
38389	0.115978	600	330	172	39	14	7	29
43461	0.147268	495	250	149	33	18	12	28
158718	0.593245	2334	1172	605	77	63	61	71
119420	0.337727	1106	581	228	35	22	11	32
23027	0.069751	208	126	99	8	2	10	8
37330	0.121874	434	297	254	16	4	26	12
36845	0.115228	315	160	159	18	5	10	15
30331	0.093734	284	180	152	13	7	11	13
60069	0.152506	419	193	217	28	9	23	27
51548	0.134844	331	212	131	18	11	15	13
20725	0.075922	306	217	143	7	5	9	7
38642	0.122439	599	372	191	18	11	16	15
60845	0.183777	1007	556	287	37	18	17	34
81534	0.239023	459	222	202	18	9	23	18
48313	0.139812	261	184	125	9	7	6	9
49184	0.139178	212	130	114	9	8	13	9
43516	0.126336	265	134	76	14	7	4	14
112047	0.34609	605	321	221	46	23	39	45
79743	0.23441	458	332	165	20	20	7	20

83345	0.237608	379	274	159	19	11	7	19
23576	0.069814	149	91	48	8	4	2	7
33632	0.091075	148	108	41	19	4	4	16
30815	0.091323	289	279	100	16	8	7	13
51444	0.145736	312	226	95	21	16	8	20
41339	0.132976	770	561	389	29	11	13	14

ncyclemvks	ncyclemvks	nfootksi19!	nfootksi20(	nfootksi20:	nmvksi199	nmvksi200	nmvksi2011
9	15	83	49	23	92	64	49
14	14	173	87	36	110	131	45
22	9	91	41	36	151	100	69
20	31	148	75	45	168	128	98
7	14	77	34	30	121	89	55
24	13	75	55	38	103	175	62
29	44	98	85	64	187	328	197
10	11	168	99	81	196	129	103
25	30	209	126	87	223	156	71
78	87	310	179	127	312	174	143
51	45	150	105	68	822	716	431
56	33	169	100	66	260	214	132
58	33	107	68	33	422	291	231
42	37	99	78	38	175	232	92
46	50	144	119	72	196	196	138
23	52	266	138	120	258	111	95
4	2	2	9	2	115	78	66
77	73	493	301	150	493	352	182
28	17	68	38	22	387	412	174
11	12	69	57	16	335	194	85
20	13	251	137	52	295	152	92
9	15	81	45	20	270	147	57
66	119	290	227	135	433	297	173
11	18	70	48	38	305	232	99
21	25	92	48	35	373	285	105
28	22	327	112	51	495	161	119
4	7	81	50	29	99	73	65
38	53	106	84	84	192	178	113
15	37	65	40	40	211	101	95
28	38	115	82	52	275	193	115
41	40	117	63	56	427	291	153
13	18	140	81	84	128	93	63
36	22	124	102	70	197	148	91
18	17	68	49	36	328	263	142
11	18	153	106	62	280	185	91
8	7	32	26	10	109	126	64
16	21	56	34	26	370	316	158
21	31	103	77	50	100	113	46
15	19	80	59	36	99	157	70
19	20	44	39	27	204	229	114
15	10	36	31	16	171	199	90
24	23	77	60	50	236	384	180
31	83	251	198	164	257	237	208
47	86	148	101	105	252	206	158
46	90	214	117	114	300	167	172
19	18	82	47	53	251	274	190

44	30	318	148	130	696	482	402
32	30	166	78	78	799	511	356
46	78	144	118	114	655	771	506
43	64	139	125	95	559	523	442
39	28	142	113	59	1080	750	286
32	39	236	132	89	1262	799	494
49	47	207	107	84	1254	857	560
17	31	76	47	42	263	195	95
14	26	99	69	57	604	421	252
21	30	322	127	91	312	178	116
10	22	159	60	59	151	94	70
69	110	710	408	215	439	338	207
13	14	237	121	80	192	104	107
19	9	238	100	70	199	141	62
27	31	259	105	68	277	160	104
28	31	192	104	56	204	119	79
21	15	237	107	59	237	127	85
24	31	124	70	51	176	120	60
32	28	251	157	68	340	208	120
13	21	164	68	32	207	106	104
50	95	738	422	323	532	494	244
12	18	154	79	63	218	160	105
27	40	186	115	84	210	169	122
42	51	236	147	82	362	312	196
20	12	164	111	73	314	282	154
35	57	195	116	110	431	287	231
18	16	191	106	63	349	210	145
60	66	438	336	192	453	508	236
15	25	218	102	68	295	181	126
27	37	391	159	113	260	136	113
19	15	188	81	45	182	95	76
16	15	139	53	30	125	56	54
35	31	372	148	88	387	167	135
79	140	1249	728	549	1262	955	581
48	42	435	215	111	671	281	182
34	31	260	139	93	360	199	149
39	35	316	203	132	388	332	161
21	17	167	67	58	449	223	109
26	19	217	131	68	349	206	114
26	35	273	135	90	382	195	126
50	56	557	297	268	677	539	301
17	28	173	84	71	291	223	164
42	42	348	201	145	580	441	247
87	111	750	472	293	1230	860	469
27	49	320	150	104	734	377	293
14	18	145	75	35	279	230	87
16	37	312	198	115	682	538	230
18	22	154	87	43	341	280	106
24	22	404	197	99	534	356	118
21	37	208	120	58	577	455	160

74	70	405	262	144	323	358	96
24	40	295	218	97	641	502	164
44	29	348	189	82	503	369	126
20	21	186	159	109	315	500	142
29	23	276	146	85	471	394	155
59	112	259	214	115	276	328	122
47	65	253	140	74	209	221	89
23	30	216	248	139	152	299	90
14	9	172	89	50	333	206	63
21	13	178	86	59	569	379	137
29	22	193	130	80	533	421	149
35	31	257	133	87	544	399	123
94	95	253	229	94	196	282	102
66	65	246	162	102	232	252	82
25	27	116	72	27	305	162	64
69	112	475	281	161	581	448	193
34	51	352	195	101	479	411	154
33	27	158	76	56	297	204	67
25	32	302	144	96	355	257	98
23	14	223	105	93	300	387	135
28	54	138	73	59	260	192	70
69	99	455	209	135	583	430	165
22	19	139	75	31	375	238	84
24	102	285	167	139	314	276	116
25	36	272	143	68	291	361	96
52	77	339	163	100	466	343	139
103	161	835	530	284	826	614	212
54	45	240	148	103	852	997	488
144	149	240	134	113	1833	1252	664
73	47	380	211	104	1405	1117	495
86	89	290	269	149	1166	1391	712
69	70	338	194	111	1794	962	628
60	66	146	129	103	889	799	452
47	72	270	236	189	1039	852	645
189	147	610	545	309	2592	2470	1379
87	100	266	155	119	1209	840	451
174	255	586	392	293	3119	2141	1473
145	126	655	408	243	2359	1988	770
151	138	831	539	329	2806	2269	1061
222	197	845	676	454	1984	1792	1183
36	37	213	133	98	1181	900	526
75	102	277	175	174	1994	1593	1056
133	89	453	255	158	2744	1593	774
102	79	440	244	163	2046	1314	674
73	93	438	233	124	3224	2054	1135
135	130	489	363	198	1939	1591	937
97	182	247	182	153	1318	1186	689
45	70	184	112	97	1196	816	493
44	46	397	169	119	1366	867	440
101	111	310	169	125	1869	1084	715

123	225	575	308	266	2457	1580	1046
82	72	324	226	101	1954	1465	717
128	143	365	279	196	1436	1194	882
63	46	266	179	79	1155	746	349
6	8	52	23	8	76	90	34
11	9	112	52	32	532	338	192
8	9	131	60	28	358	201	103
4	3	86	42	21	145	76	41
2	8	70	27	12	145	141	66
3	11	84	35	13	170	85	28
0	0	32	8	1	58	47	22
13	14	169	75	32	256	178	109
28	21	264	161	75	492	555	240
21	9	144	76	36	1016	694	290
8	3	129	55	28	120	57	44
8	5	50	27	22	149	127	57
8	3	42	21	22	185	157	84
7	4	166	65	37	349	158	68
3	2	18	9	5	104	55	25
12	10	108	62	30	726	466	232
7	13	62	23	22	372	305	199
9	8	121	59	30	357	197	93
18	15	331	144	79	649	428	162
10	11	81	38	20	259	269	145
11	21	246	79	128	216	124	148
14	13	98	55	39	765	499	568
18	3	80	34	15	484	323	183
79	90	549	342	196	549	335	203
24	23	261	102	56	362	212	94
9	7	120	65	29	141	82	36
11	16	81	59	44	244	179	127
14	7	96	57	25	429	242	127
18	11	267	103	77	190	125	57
61	58	1412	630	327	822	466	211
20	11	443	208	76	603	337	133
1	10	26	16	15	173	107	71
2	20	66	31	34	336	251	193
5	8	64	19	29	224	131	118
7	11	68	38	30	198	132	106
8	23	81	26	31	300	152	160
8	11	68	46	36	234	148	77
3	7	35	25	16	255	183	115
10	11	86	42	37	475	309	126
16	15	164	63	45	768	454	220
9	23	164	71	65	274	137	111
7	6	45	43	31	204	132	88
8	13	81	32	16	116	88	82
7	4	52	17	25	191	109	45
23	38	221	121	71	331	171	111
20	7	158	89	56	270	198	96

9	7	127	73	48	230	181	101
4	2	54	32	10	86	51	34
4	4	40	24	16	83	79	21
7	7	50	29	11	213	227	80
16	8	129	65	26	156	140	59
10	11	74	50	29	630	479	324
